# Parthenolide Induces Apoptosis and Cell Cycle Arrest of Human 5637 Bladder Cancer Cells *In Vitro*

**DOI:** 10.3390/molecules16086758

**Published:** 2011-08-09

**Authors:** Guang Cheng, Liping Xie

**Affiliations:** Department of Urology, The First Affiliated Hospital, School of Medicine, Zhejiang University, Hangzhou, Zhejiang 310003, China

**Keywords:** bladder cancer, parthenolide, cell cycle arrest, apoptosis

## Abstract

Parthenolide, the principal component of sesquiterpene lactones present in medical plants such as feverfew (*Tanacetum parthenium*), has been reported to have anti-tumor activity. In this study, we evaluated the therapeutic potential of parthenolide against bladder cancer and its mechanism of action. Treatment of bladder cancer cells with parthenolide resulted in a significant decrease in cell viability. Parthenolide induced apoptosis through the modulation of Bcl-2 family proteins and poly (ADP-ribose) polymerase degradation. Treatment with parthenolide led to G1 phase cell cycle arrest in 5637 cells by modulation of cyclin D1 and phosphorylated cyclin-dependent kinase 2. Parthenolide also inhibited the invasive ability of bladder cancer cells. These findings suggest that parthenolide could be a novel therapeutic agent for treatment of bladder cancer.

## 1. Introduction

Bladder cancer is the fourth most common cancer in the USA, with an expected 71,000 newly diagnosed cases and 14,300 deaths [1] in 2009. It has the highest lifetime treatment cost of any cancer because of the need for frequent interval cystourethroscopy, urine cytology, and radiological evaluations. Despite recent advances in surgical and chemotherapeutic procedures, the 5-year survival rate in patients with invasive and metastatic bladder cancer remains very low [2]. Therefore, other effective strategies are clearly needed to improve the survival of these patients.

Parthenolide (PTL), a sesquiterpene lactone isolated from feverfew (*Tanacetum parthenium*), is an herbal medicine that has been used traditionally for the relief of migraine [3], inflammation [4] and rheumatoid arthritis [5]. Recently it has been shown to exhibit anti-cancer activities in a wide variety of solid tumors by inducing apoptosis and necrosis [6,7,8]. In particular, PTL has been proven to be able to preferentially eradicate acute myelogenous leukemia stem and progenitor cells, while sparing normal hematopoietic cells [9]. The biological activities of PTL have been ascribed to the inhibition of DNA binding of transcription factors NF-κB and STAT-3, reduction in MAP kinase activity and the generation of reactive oxygen [10,11,12]. The objectives of the present study were to investigate the role of PTL on targeting bladder cancer cells. Our results demonstrated PTL was cytotoxic to 5637 bladder cancer cells *in vitro*, thereby representing a promising therapeutic agent for treatment of bladder cancer.

## 2. Results and Discussions

### 2.1. Cytotoxicity Effects of PTL on 5637 Bladder Cancer Cells

**Figure 1:**
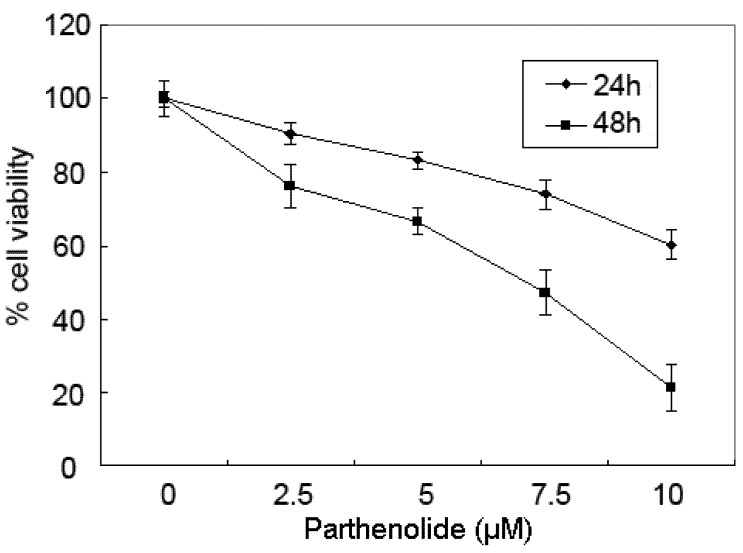
Parthenolide inhibited cell viability of human bladder cancer 5637 cells in a dose- and time-dependent manner. Viability of cells was determined by the MTT assay. Reduced cell viability was observed with parthenolide treatment (2.5–10 μM) concentrations at 24, 48 h. The data are presented as means ± SD.

The cytotoxic effect of PTL on human bladder cancer 5637 cells was determined with varying concentrations (0–10 μM) and times (24–48 h) by the MTT assay. As shown in Figure 1, the survival rate of 5637 cells decreased markedly with incremental doses of PTL up to 10 μM. The survival curves shifted to the right with longer drug exposures. These data indicate that PTL exerts a significant cytotoxic effect upon 5637 cells in a dose- and time-dependent manner.

### 2.2. Induction of Apoptosis of 5637 Cells by PTL

**Figure 2:**
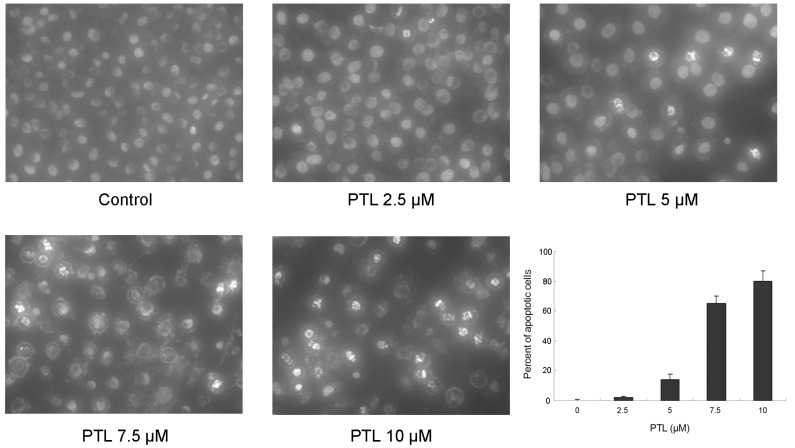
Detection of apoptosis after exposure to parthenolide for 48 h in 5637 bladder cancer cells. Cells were stained with Hoechst stain (H33258), visualized by UV microscopy, and quantitated by counting condensed and fragmented nuclei in five randomly selected areas. The proportion of apoptotic cells was expressed as a percentage.

We investigated whether the reduced cytotoxicity induced by PTL was due to induction of apoptosis. Changes in nuclear morphology were evaluated after Hoechst staining using fluorescence microscopy. As shown in Figure 2, high concentrations of PTL treatment led to significant chromatin condensation or fragmentation, which correlated with the occurrence of apoptosis. The degree of apoptosis was calculated by counting the proportion of cells with condensed chromatin, showing that treatment of 5637 cells with PTL resulted in a dose-dependent induction of apoptosis.

### 2.3. PTL-Induced Apoptosis Mediated by Bcl-2 Family Modulation and PARP Cleavage

**Figure 3:**
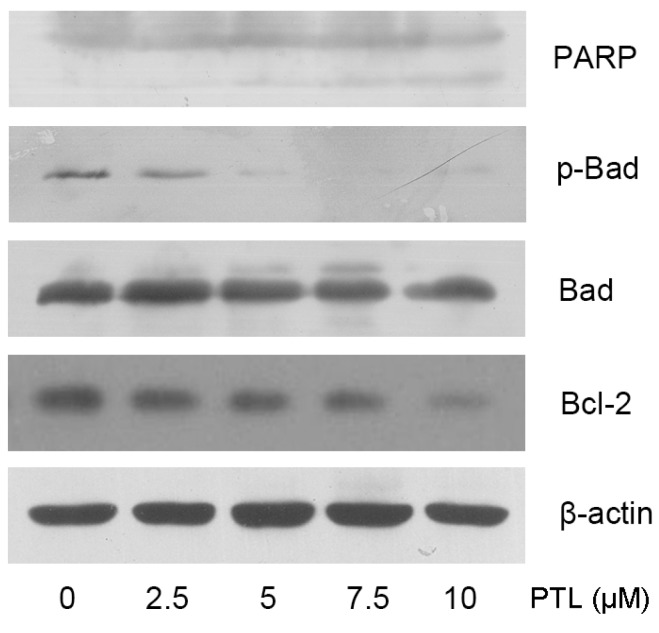
Parthenolide treatment activated PARP and modulated expression of apoptotic genes in 5637 bladder cancer cells.

To gain further insight into the way in which PTL is cytotoxic, we investigated several key molecules known to regulate apoptosis. It has been demonstrated that the proteolytic cleavage of PARP represents one of the final steps of the proteolytic caspase cascade and reliably indicates ongoing apoptosis [13]. Western blotting analysis indicated that treatment of 5637 cells with PTL resulted in a dose-dependent activation of PARP 48 h after PTL treatment. Next, we examined the effect of PTL treatment on Bcl-2 family proteins in 5637 cells. We observed that the expression of phosphorylated Bad and the anti-apoptotic Bcl-2 was downregulated, which is indicative of induction of apoptosis (Figure 3).

### 2.4. Cell Cycle Accumulation at G1 Phase after Treatment with PTL

**Figure 4:**
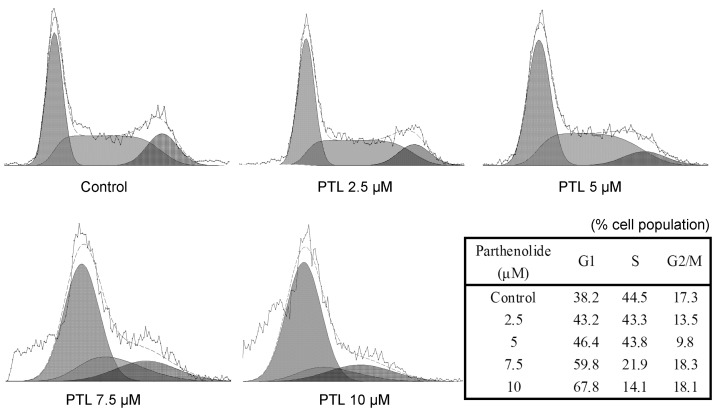
Cell cycle analysis of 5637 bladder cancer cells treated with parthenolide. Cells were cultured with different concentrations of parthenolide for 24 h and then stained with propidium iodide. The DNA content was analyzed by flow cytometry. G1, S, and G2/M indicate cell cycle phases.

Since the induction of apoptosis might be mediated through the cell cycle arrest, we next examined the effect of PTL on cell cycle perturbations. As shown in Figure 4, PTL treatment was found to result in a significant dose-dependent increase of cell population in the G1 phase of the cell cycle in 5637 cells. The increase in cell population in the G1 phase was found to be associated with a concomitant decrease in cell population in the S phase, whereas the population of cells in G2/M phase did not change significantly as compared with the corresponding controls.

### 2.5. Modulation of Cell Cycle related Proteins by PTL

**Figure 5:**
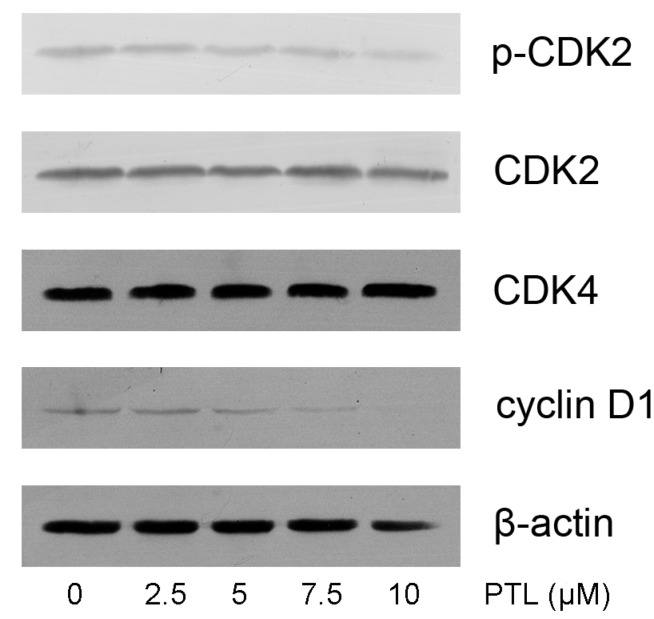
Effects of parthenolide cell cell cycle related proteins in the G1 phase.

Because our results indicated that PTL treatment caused G1 phase cell cycle arrest, we further examined the effect of PTL on cell cycle related proteins in the G1 phase. We assessed cyclin D1, CDK2 and CDK4, the key regulators of G1–S phase transition. Using Western blotting analysis, we found that PTL treatment resulted in a significant dose-dependent reduction of cyclin D1, but no change in CDK4 levels. We also found that PTL treatment of 5637 cells leads to a dose-dependent decrease phosphorylation of p-CDK2, which is important for the G1 to S transition (Figure 5).

### 2.6. Inhibition of Cell Invasion by PTL

**Figure 6:**
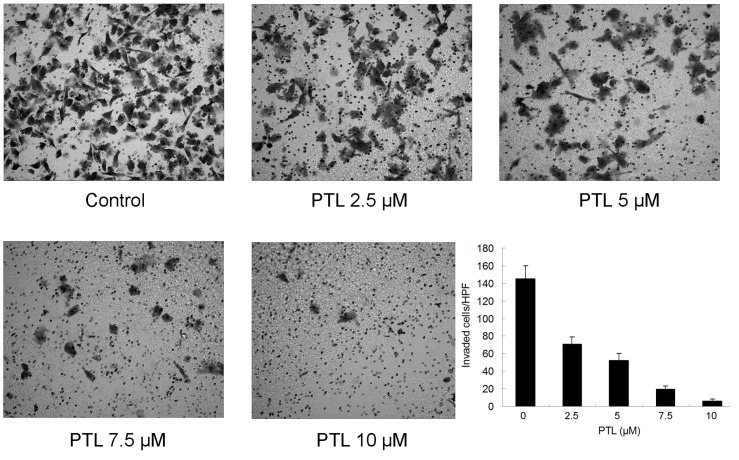
Effect of parthenolide on 5637 cell invasion. Cells were treated with different concentrations of parthenolide for 48 h, then were fixed and stained and counted. The invaded cells were expressed as a percentage.

We next sought to determine whether saRNA could inhibit invasion of bladder cancer cells *in vitro.* Matrigel invasion assays were performed to examine whether invasion of the 5637 cells treated with PTL were inhibited. As shown in Figure 6, the numbers of cells that passed through the filter and into the lower chamber were obviously reduced after treated with PTL at different concentrations, exhibiting a significant decrease in invasive ability as compared to Control.

Bladder cancer is one of the leading causes of cancer death throughout the world, and the five-year relative survival rates are about 6% if people are diagnosed with bladder cancer at a distant stage. Chemotherapy continues to be the treatment of choice for advanced/metastatic bladder cancer with response rates as high as 70% [14]. However, in addition to the side effects in general, the efficacy of chemotherapy is often hampered by drug resistance. Therefore, there is a need to identify novel agents active against bladder cancer, particularly for treatment of the poor prognosis patients who are less likely to respond to conventional drugs.

Recent studies have identified PTL as a promising agent against certain cancers. In the present study, we aimed to characterize PTL’s anticancer activity in bladder cancer. Consistent with earlier observations, we found that PTL inhibits the proliferation and viability of bladder cancer cells by inducing apoptosis, as evidenced by PARP cleavage and nuclear morphology. It is suggested that the apoptosis of 5637 cells induced by PTL might act by modulation of Bcl-2 family proteins and probably followed by activation of caspase-3 and caspase-9, which play a central role in apoptosis, resulting in degradation of PARP. In a previous investigation, Shanmugam *et al.* have shown that DMAPT, a parthenolide derivative, exerts an apoptotic effect on bladder cancer cells [15]. Our study demonstrated that PTL itself is also able to induce cell death in bladder cancer cells.

The cell cycle and apoptosis are intimately related, and together play an important role in the sensitivity of cancer cells to chemotherapy [16]. In this study, we investigated the cell cycle distribution after treatment with PTL and found that PTL induced cell cycle arrest at G1 phase. However, previous studies has demonstrated that PTL induced accumulation of cancer cells in S or G2/M phase [17,18,19], and this could be explained by the possibility that PTL causes alteration of cell cycle in a cell type-dependent manner. p53, in response to DNA damage, triggers a variety of cell cycle-regulatory events to limit the proliferation of damaged cells [20]. In our experiments, we used 5637 cells, which harbor two TP53 mutations, at codon 72 (Arg > Pro) and codon 280 (Arg > Thr) [21]. It has become increasingly clear that many mutant p53 forms not only lose the wild-type p53 tumor suppressor activity and acquire dominant-negative activities, but also possesses a “gain-of-function” activity, which ranged from enhanced cell proliferation, to increased tumorigenicity *in vivo* [22]. Therefore, it is important to further study the role of mutant p53 in parthenolide-induced cell cycle arrest.

The molecular mechanisms of proapoptotic action of PTL on cancer cells have been described. On the transcriptional level, PTL inhibits both NF-κB and STATs transcription factors activity, resulting in the up- or down-regulation of specific genes related to apoptosis [11,12]. 5637 cell line expresses a high level of cyclooxygenase-2 (COX-2), which is demonstrated to correlate with poor differentiation, increased tumor size, more metastasis and decreased overall survival [23]. PTL has been reported to be a COX-2 inhibitor, thus COX-2 might play a key role in parthenolide-induced apoptosis in 5637 cells. In addition, PTL may induce ROS generation and intrinsic apoptotic pathway [10]. Importantly, several studies have showed that PTL specifically affects malignant but is harmless to normal cells [9,24,25].

Tumor invasion and metastasis are a multistep process that includes cell proliferation, proteolytic degradation of the extracellular matrix, cell migration through the basement membranes to reach the circulation system and the remigration and growth of tumors at metastatic sites [26]. In this study, we demonstrated that PTL effectively suppressed invasive phenotype of bladder cancer cells, although the reduced cell invasion might be partly due to apoptosis or proliferation inhibited by PTL. Liu *et al.* has also shown that PTL inhibited BxPC-3 pancreatic cancer cell migration and invasion *in vitro* [27].

However, despite its broad *in vitro* anti-cancer activity, the solubility of PTL is relatively poor, making pharmacologic use of the compound difficult. Curry *et al.* [28] showed that 4 mg of parthenolide, given daily as part of the feverfew administered as an oral tablet, does not provide detectable plasma concentrations. In contrast, parthenolide derivatives seem to reach much higher plasma levels than the parent agent [29]. Therefore, the new routes of administration of parthenolide leading to high tissue concentrations in target organ should be explored and the potential biological activity of parthenolide metabolites should be considered for future investigations.

## 3. Experimental

### 3.1. Cell Culture and Reagents

Bladder cancer cell line 5637, which originates from a grade 2 bladder transitional cell carcinoma, and contains a mutant p53 gene, was obtained from the Institute of Biochemistry and Cell Biology, Chinese Academy of Sciences (Shanghai, China). The cells were cultured in RPMI 1640 medium supplemented with 10% fetal bovine serum in a humidified atmosphere containing 5% CO_2_ maintained at 37 °C. PTL and MTT were purchased from Sigma Chemical Co. (St. Louis, MO, USA). Antibodies used in this study were obtained from Santa Cruz Biotechnology (Santa Cruz, CA, USA) and Cell Signaling Technology (Beverly, MA, USA).

### 3.2. Cell Viability Assay

The effect of PTL on the viability of 5637 cells was evaluated by the MTT assay. Approximately 1 × 10^4^ cells were seeded in each well of a 96-well plate. After overnight incubation, the cells were treated with different concentrations of PTL (0–10 μM) in DMSO. After incubation for the indicated time, the MTT reagent (20 μL of 5 mg/mL solution) was added to each well and incubated at 37 °C for 4 h. The plates were spun, and the purple colored formazan precipitates were dissolved in DMSO (150 μL). Absorbance was measured at 490 nm using the MRX II absorbance reader (DYNEX Technologies, Chantilly, VA, USA). Results were expressed as a percentage of growth, with 100% representing control cells treated with DMSO alone.

### 3.3. Nuclear Morphology

Cells were seeded in 24-well plates and exposed to PTL treatment for 48 h. After washing twice with PBS, the cells were stained with Hoechst 33342 solution (final concentration 10 mg/mL) and then incubated at 37 °C for 10 min. The nuclear morphology was observed using ultraviolet light under an OLYMPUS microscope.

### 3.4. Cell Cycle Distribution Analysis

Cells were seeded in 6-well plate and incubated overnight before treatment. After harvest at 24 h following treatment, cells were washed twice with pre-chilled PBS and resuspended in PBS (100 μL) at a concentration of 1 × 10^6^ cells/mL. Cell cycle analysis was performed using the Coulter DNA PrepTM Reagents Kit (Beckman Coulter, Fullerton, CA, USA). Finally cell cycle analysis was performed by Beckman Coulter FC500 Flow Cytometry System with CXP Software (Beckman Coulter) within 1 h and the raw data was analyzed by Multicycle for Windows (Beckman Coulter).

### 3.5. In Vitro Cell Invasion Assay

A Transwell cell culture chamber (Millipore, Bedford, MA, USA) with PET membranes (24-well insert, 8-μm pore size, Millipore) was coated with Matrigel, dried and reconstituted at 37 °C with culture medium. Medium (0.6 mL) supplemented with 20% fetal bovine serum was added to each well of the plate to act as a chemoattractant in the lower chamber. Then the cells were suspended at a concentration of 4 × 10^5^ cells/mL in medium with different concentrations of PTL, and 0.2 mL of each was added to the top chamber. Cells were incubated for 48 h, and those that did not migrate through the pores were removed by scraping the upper surface of the membrane with a cotton swab. Cells that had migrated to the lower surface of the membrane were fixed for 5 min in 100% methanol and stained with 0.1% crystal violet for 2 min. The cells that invaded through the insert were counted in five random fields and expressed as the average number of cells per field. These experiments were done in triplicate and performed a minimum of three times.

### 3.6. Western Blot Analysis

Cells were harvested at 48 h following PTL treatment, washed, and lysed with lysis buffer (10 mmol/L Tris-HCl, 0.25 mol/L sucrose, 5 mmol/L EDTA, 50 mmol/L NaCl, 30 mmol/L sodium pyro-phosphate, 50 mmol/L NaF, 1 mmol/L Na_3_VO_4_, 1 mmol/L PMSF, and 2% cocktail). Protein concentration in the resulting lysate was determined using the bicinchoninic acid protein assay. Appropriate amounts of protein were separated by electrophoresis in 8%–12% Tris-glycine polyacrylamide gels and transferred to nitrocellulose membranes. Membranes were blocked then incubated overnight with the appropriate primary antibody at dilutions specified by the manufacturer. They were next washed and incubated with the corresponding HRP-conjugated secondary antibody at 1:2,500 dilution in Tris-buffered saline. Bound secondary antibody was detected using an enhanced chemiluminescence system (Pierce Biotechnology Inc., Rockford, IL, USA).

### 3.7. Statistical Analysis

Data are presented as the mean ± standard deviation (SD). One-way analysis of variance (ANOVA) was used to determine significance among groups. A value of *p* < 0.05 was considered to be significant.

## 4. Conclusions

In summary, our results suggested that parthenolide may have powerful activity against bladder cancer, and should be considered as potent candidate to facilitate anticancer treatment if further positive studies *in vivo* are confirmed.
